# 

**Published:** 1860-03

**Authors:** 


### CONTENTS.

ORIGINAL ESSAYS AND COMMUNICATIONS.

Superficial Decay. By GEO. S. FOUK, ... 393

Pray for Me ... 399

Valedictory Address, ... 409

Reply. By L. H. HENDRICK, M. D., D. D. S., ... 425

PROCEEDINGS OF SOCIETIES.

Discussions of the Michigan State Dental Association, ... 429

Proceedings of Pennsylvania Society of Dental Surgery ... 438

SELECTIONS.

New York Academy of Medicine, ... 440

CORRESPONDENCE, ... 447

EDITORIAL, ... 451

